# Difficulties Encountered by People With Depression and Anxiety on the Web: Qualitative Study and Web-Based Expert Survey

**DOI:** 10.2196/12514

**Published:** 2019-10-31

**Authors:** Renaldo Bernard, Carla Sabariego, Alarcos Cieza

**Affiliations:** 1 Chair for Public Health and Health Services Research, Research Unit for Biopsychosocial Health Department of Medical Informatics, Biometry and Epidemiology Ludwig-Maximilians-Universität München Munich Germany; 2 Blindness and Deafness Prevention, Disability and Rehabilitation World Health Organization Geneva Switzerland

**Keywords:** World Wide Web, depression, anxiety, accessibility, interview, persona, expert study, eHealth, usability, user experience, facilitators, barriers, mental disorders

## Abstract

**Background:**

Depression and anxiety are the most common mental health conditions, and they were identified as leading contributors to global disability in 2016. People with these conditions rely on Web-based resources as a source of accurate health information, convenient and effective treatment, and essential social support. However, a recent systematic review revealed several potentially limiting difficulties that this group experiences online and also suggested that there is a partial understanding of these difficulties as only difficulties associated with neurocognitive, but not sociocognitive, deficits were identified. Therefore, this study fills this knowledge gap and contributes to a more robust and fuller understanding of the difficulties this group experiences online.

**Objective:**

The objective of this study was to identify the difficulties people with depression and anxiety experience when using the Web and the Web activities that are most associated with the experience of difficulties.

**Methods:**

The study employed data triangulation using face-to-face semistructured interviews with 21 participants affected by depression and anxiety and a comparison group (7 participants) without mental disorders (study 1) as well as a persona-based expert online survey with 21 mental health practitioners (MHPs) who treated people with depression and anxiety (study 2). Framework analysis for both studies proceeded through 5 stages: (1) familiarization, (2) identifying a thematic framework, (3) indexing, (4) charting, and (5) mapping and interpretation.

**Results:**

In study 1, 167 difficulties were identified from the experiences of participants in the depression and anxiety group were discussed within the context of 81 Web activities, services, and features. From these, 4 themes and 12 subthemes describing the difficulties people with depression and anxiety experienced online were identified. Difficulties relating to the subtheme lack of control over access and usage were the most common difficulties experienced by participants in the depression and anxiety group (19/21). Sixteen difficulties identified from the experiences of participants in the comparison group were discussed within the context of 11 Web activities, services, and features. Most participants in the comparison group (6/7) contributed to the subtheme describing difficulties with unexpected and irrelevant content. In study 2, researchers identified 3 themes and 10 subthemes that described the perceived difficulties people with depression and anxiety might experience online as reported by MHPs. Practitioners linked these difficulties with 22 common impairments, limitations in activities of daily life, and diagnostic criteria associated with depression and anxiety.

**Conclusions:**

People with depression and anxiety also experience difficulties when using the Web that are related to the sociocognitive deficits associated with their conditions. MHPs have a good awareness of the difficulties that people with depression and anxiety are likely to experience when using the Web. This investigation has contributed to a fuller understanding of these difficulties and provides innovative guidance on how to remove and reduce them for people with depression and anxiety when using the Web.

**International Registered Report Identifier (IRRID):**

RR2-10.1007/978-3-319-21006-3_3

## Introduction

### Background

Depression and anxiety are the most common mental health conditions, and they were identified as leading contributors to global disability in 2016 [1]. Among other resources, people with depression and anxiety often rely on the Web as a resource for health information gathering [2,3], a source of convenient and effective treatment across the life span [4,5], and as a means to connect with others and receive social support [6].

However, a recent systematic review revealed that people with mental health conditions experience difficulties when using the Web [7] that might limit how much they benefit from the Web and that are also poorly understood. The review highlighted a narrow range of difficulties solely related to neurocognitive dysfunction, that is, impaired attention, processing and responding to information slowly, and problem solving. Although sociocognitive deficits, that is, impaired affect regulation and difficulty processing emotional cues, are also as important features of mental health conditions as neurocognitive deficits [8,9], no difficulties relating to sociocognitive deficits were identified by the 13 included studies. The review suggests that despite a relatively recent surge of interest within the general field of human-computer interaction into sociocognitive phenomena [10], this trend seemingly does not apply to Web accessibility research focused on people with mental health conditions. For example, the included studies did not employ methods that would unearth possible difficulties related to sociocognitive deficits, and other researchers have arrived at similar conclusions [11].

Therefore, this investigation will provide a broader perspective that could fill the abovementioned knowledge gap and contribute to a more robust and fuller understanding of the difficulties people with depression and anxiety experience when using the Web. This knowledge will primarily assist Web professionals in creating more accommodating experiences for people with depression and anxiety online and allow this group better access to the opportunities available to everyone else using the Web. It is also expected that the Web would benefit from greater inclusivity where people with depression and anxiety could also make valuable contributions to this informational resource. This research was conducted under the BETTER (weB accEssibiliTy for people wiTh mEntal disoRders) project that investigates Web accessibility for people with depression and anxiety and focuses on these conditions because of their high burden relative to that of other mental health conditions [12].

### Objectives

The objective of this study was to identify the difficulties people with depression and anxiety experience when using the Web and the Web activities for which the most difficulties are reported. This study specifically aimed to achieve its objective through triangulation [13] using 2 data sources: face-to-face interviews with people with depression and anxiety and a comparison group without mental disorders (study 1) as well as a persona-based expert Web-based survey with mental health practitioners (MHPs) who treated people with depression and anxiety (study 2).

## Methods

### Study Designs

Semistructured interviews with people with depression and anxiety and a comparison group were conducted in study 1, and an MHP expert online survey was conducted in study 2. Data triangulation [13] was used to add breadth and depth to the analysis and to evaluate the robustness of findings. Although useful for strengthening the study’s conclusions and reducing the risk of false interpretations, triangulation was not used to reduce the findings to a single common truth or for validating one view with another view. Of all the stakeholders in the care of people with depression and anxiety, the person themselves and their therapists were the most accessible and well-suited sources of insight available to this investigation. Study 1 and 2 data were collected, aggregated, and later discussed.

### Study 1—A Semistructured Interview Study With a Comparison Group

#### Recruitment

Participants were recruited using purposive sampling, first with the aim of maximum variation [14,15], based on age [16,17], gender [18], and condition severity [19] as these factors have been found to influence Web usage. However, as this recruitment strategy proved difficult to acquire participants overtime, potential participants who met the inclusion criteria were later considered for participation as well. Participants were recruited using posters placed on message boards around the university and short recruitment messages broadcasted via the university’s intranet news feed and social media accounts. Participants were recruited in the United Kingdom based on the following criteria: they were aged ≥18 years, skilled Web users, diagnosed with depression and or an anxiety disorder, and had no sensory or physical impairments that required the use of adaptive or assistive technologies to operate a computer system. It was important only to include skilled Web users to reduce the likelihood that difficulties encountered could be associated with being an unskilled user rather than the targeted conditions. Participants in the comparison group were recruited to match the demographic profile of participants with a diagnosis, but with 1 exception, these participants were encouraged by recruitment advertisements and the participant information sheet to only consider participating if they were never diagnosed with a mental disorder. Data saturation (ie, no new data, themes, and coding) [20] determined the final number of participants to recruit. Data saturation helps to ensure that the study is supported by adequate and quality data [21].

Screening tools were used to ensure that participants met the inclusion and exclusion criteria. Potential participants in both groups scoring more than 25 on the 10-item abbreviated Web use skills index for the general population [22] were invited to participate. Beck Depression Inventory-II [23] and Beck Anxiety Inventory [24] measure symptom severity at 3 levels (ie, mild, moderate, and severe) and were used for this purpose. Those in the comparison group were assessed for depression and anxiety using the Patient Health Questionnaire for Depression and Anxiety (score between 0 and 2), which has demonstrated high sensitivity and specificity in screening for both conditions [25] and has much fewer items than the other 2 instruments.

#### Data Collection

Ethical approval was granted by the ethics committee of the University of Southampton. Those who passed screening and gave written consent were invited to participate in a face-to-face semistructured interview lasting between 60 and 90 min. Semistructured interviews allowed researchers to gather rich descriptive data about the experiences of participants when using the Web. The method is also useful for exploring this research domain that is in its infancy [26]. Furthermore, it allows for the flexibility to pursue unexpected experiential paths as shared by the participants without losing focus on the key issues of investigation [26]. A topic guide (Multimedia Appendix 1) was used to ask questions about the difficulties participants experienced when using the Web during their daily lives. The interviews were conducted in private rooms around the university between June and November 2016 and were transcribed verbatim from digital audio recordings and evaluated for accuracy before being analyzed. Personally identifiable data were removed from transcriptions, and pseudonyms were used for participants.

#### Data Analysis

Framework analysis as outlined by Ritchie et al [27] is commonly used to analyze stakeholder accounts from in-depth semistructured interviews. Although the technique primarily subscribes to a thematic approach, it also permits identified themes from semistructured interview narratives to be organized around research questions [28]. Aided by VERBI’s MAXQDA 12 qualitative research software package, researchers proceeded through 5 stages: (1) familiarization, (2) identifying a thematic framework, (3) indexing, (4) charting, and (5) mapping and interpretation.

Researchers became familiar with collected data by listening to the recordings and reading and rereading transcripts while progressively making initial notes of any thoughts that surfaced. Themes were then identified and questioned. Data were sifted, and selected quotes were sorted and later rearranged thematically [29]. The discovered themes were compared to ensure they accurately reflected the data. The analysis then went into a deeper interpretative phase focusing on extracts that illuminated participants’ accounts in vivid detail.

### Study 2—A Mental Health Practitioner Expert Online Survey Study

#### Recruitment

Purposive sampling, specifically expert sampling [30,31], was used to recruit participants for this survey. Respondents had to be aged ≥18 years; had to be an accredited, a chartered, or a registered member of a professional body in the United Kingdom for MHPs; and must have had experience treating people with depression and anxiety. MHPs were considered suitable experts for this study as they aim to improve their patients’ mental health through therapy that benefits from a deep understanding of their patients’ lived experiences [32]. Therefore, it was expected that MHPs would have a good understanding of the difficulties this group might encounter online as well. We examined the potential difficulties people with depression and anxiety face on the Web, as explained by MHPs included in an online database directory of MHPs between January and October 2016. Data saturation [20] determined the final number of respondents. The Checklist for Reporting Results of Internet E-Surveys [33] for this survey is presented in Multimedia Appendix 2.

#### Data Collection

Ethical approval was granted by the ethics committee of the University of Southampton. Respondents gave their consent before participating in the Web-based survey, which was conducted between January and October 2016. They then answered questions relating to 2 of the 4 personas that were randomly given. One persona focused on depression and the other on an anxiety disorder.

The survey asked demographic questions (eg, educational background and expertise) and open-ended questions about the personas that were provided (Multimedia Appendix 3). The 4 personas used were fictional characters (2 with depression and 2 with anxiety) developed for this study by RB based on information about impairments, activity limitations and participation restrictions experienced by people with depression and anxiety, diagnostic criteria associated with these conditions, and also scenarios that featured a wide range of common Web activities.

The International Classification of Functioning, Disability, and Health Core Set for Depression is an internationally accepted and evidence-based selection of functioning domains [34] that covers the spectrum of symptoms and limitations in the functioning of persons with depression. The seminal study on horizontal epidemiology [35] involving systematic literature reviews, content analysis of patient-reported outcomes and outcome instruments, clinical input, and a qualitative study generated a useful group of psychosocial difficulties commonly experienced across brain disorders. The International Statistical Classification of Diseases and Related Health Problems 10th edition is a classification created and maintained by the World Health Organization [36]. The Web activity taxonomy developed by Sellen et al [37], and that later received strong support from Kellar et al [38], was used to animate personas presented to respondents. Feedback on the first version of the survey was obtained from 2 MHPs who participated in the survey pilot, and some modifications were made as a result.

#### Data Analysis

The data analysis technique used on the data from the semistructured interviews was also applied to these data.

## Results

### Summary of Results

A total of 167 difficulties that people with depression and anxiety experienced when using the Web were identified in study 1, and 10 difficulties were identified in study 2. A comparison of these findings will be shared after the findings for each study are detailed below.

### Study 1—Semistructured Interview Study With a Comparison Group

This study had a sample of 28 participants (8 males and 20 females) aged between 18 and 69 years (Table 1). A total of 16 females and 5 males were recruited for the sample of people with depression and anxiety. Moreover, 4 females and 3 males were included in the comparison group. An independent samples *t* test was conducted to compare the level of Web skill in the depression and anxiety group with that in the comparison group. There was no significant difference in the level of Web skill in the depression and anxiety (mean 40.10, SD 6.94) group and comparison (mean 41.14, SD 9.26) group (*t*_26_=0.32; *P*=.75, 2-tailed). A comparison of the identified difficulties encountered by participants in both groups reveals that they have noticeably different experiences when using the Web.

**Table 1:** Sample demographics of the semistructured interview study with a comparison group.

Characteristics		Depression and anxiety group (n=21), n	Comparison group (n=7), n
**Sex**			
	Male	5	3
	Female	16	4
**Age (years)**			
	18-29	11	5
	30-49	7	1
	50-69	3	1
**Condition**			
	Anxiety	2	—^a^
	Depression	9	—
	Anxiety and depression	10	—
	None	—	7
**Condition severity: depression**			
	Mild	2	—
	Moderate	6	—
	Severe	11	—
**Condition severity: anxiety**			
	Mild	0	—
	Moderate	5	—
	Severe	4	—
**Frequency of Web usage**			
	Several times a day	20	6
	Once a day	1	1
	Several times a week	0	0
	Once a week	0	0
	Once a month or less	0	0

^a^Not applicable.

The 167 difficulties identified from the experiences of participants in the depression and anxiety group were discussed within the context of 81 Web activities, services, and features. The majority of participants in the depression and anxiety group reported difficult experiences that were captured in each theme (Table 2).

The 16 difficulties identified from the experiences of participants in the comparison group were discussed within the context of 11 Web activities, services, and features. These difficulties were often encountered with momentary negative affect and, occasionally, resulted in dislike for the particular Web activity, service, or feature. Difficult experiences shared by most participants in the comparison group were represented by theme 1 (Table 2). Each of the remaining 3 themes included difficult experiences discussed by a small number of participants in this group (Table 2).

**Table 2:** Number of participants in the people with depression and anxiety group compared with number of participants in the comparison group by theme.

Theme	Participants in the people with depression and anxiety group (n=21), n	Participants in the comparison group (n=7), n
Inappropriate and sensitive content	20	6
Lack of safety, privacy, and security controls	20	4
Lack of adequate support	19	1
Difficult user interfaces	17	3

#### Theme 1: Inappropriate and Sensitive Content

A total of 4 subthemes were identified within this theme: (1) unexpected, irrelevant, and inappropriate content is upsetting; (2) reminders of upsetting experiences and negative affect triggers; (3) social comparison cues on social media that result in increased negative affect; and (4) abusive content limits Web usage by those who avoid it. The majority of participants, 20 out of 21 in the depression and anxiety group and 6 out of 7 in the comparison group, identified inappropriate content as a source of difficulty under this theme. Other subthemes were only reported by participants in the depression and anxiety group who discussed difficulties with sensitive and abusive content (Table 3).

#### Unexpected, Irrelevant, and Inappropriate Content Is Upsetting

Exposure to inappropriate content was followed by feelings of upset, frustration, and helplessness. This negatively impacted the ability of some participants in the depression and anxiety group to complete tasks for up to a day:

See a photo and it’s affected my mood for the rest of the day. I’ll be there sat when I go to work, flip through social media, all of a sudden something hits, feel low, go to work and it doesn’t pick up and then I can’t perform at work and then I get sent home which makes me feel even worse.

Shane, depression and anxiety group

Participants in the comparison group generally felt upset by inappropriate content but saw such content as being a regular part of using the Web and appeared better able to quickly overcome these feelings than those in the depression and anxiety group:

Was it upsetting for a long time or was it just that moment?

Moderator

Just that initial moment.

Marita, comparison group

#### Reminders of Upsetting Experiences and Negative Affect Triggers

Exposure to sensitive content resulted in involuntarily recollecting memories of personally meaningful issues that were upsetting for a temporary or prolonged period.

Such sensitive content on the Web is varied and diverse, as shown in Table 3. Some types of sensitive content are also composed to provoke a strong negative emotional response deliberately, for example, graphic content used in news stories and promoted posts on social media related to appeals by causes supporting people and animals in need:

It’s almost like some adverts I can’t watch because I just think, “I mean, I know they are poor, starving children in Africa”...I’m paying you know, and I’m doing [my] bit. But I literally get to the point, I sit and think, “Oh my God, if that was me, if that was my child,” I mean, I would just give away, I could never, I’d just be giving away my clothes.

Clara, depression and anxiety group

No participant in the comparison group reported a similar difficulty.

**Table 3:** Difficulties with sensitive and inappropriate content experienced by participants in both sample groups by subtheme.

Subtheme—number of participants from DA^a^ (n=21) and comparison (n=7) groups	Difficulty reported by the DA group participants	Difficulty reported by the comparison group participants
Unexpected, irrelevant, and inappropriate content is upsetting (DA group: 17/21 and comparison group: 6/7)	Exposure to upsetting offensive content from social connections (eg, violence, trifle, overshare, exaggeration, and constant help seeking), news websites (eg, violence, headline marquee, articles, and political bias), and advertising (eg, prominently positioned, excessive amounts on page, disguised, misleading, obstructive, persistent, distracting, and intrusive) Notifications highlighting insignificant information on social media platforms Unexpected and inappropriate search results	Exposure to offensive content and personally critical comments from social connections, online dating counterparts (eg, sexual content and inappropriate contact), news websites (eg, untrustworthy articles, political bias, and violent acts), and advertising Notifications from social media platforms
Reminders of upsetting experiences and negative affect triggers (DA group: 14/21 and comparison group: 0/7)	Inappropriate help-seeking behavior on social media by those with similar negative experiences Social media features—highlighting content such as status updates, images, and posts from social connections from current date in the past—that trigger memories of upsetting experiences Personally relevant content (eg, status updates, images, posts from friends, adverts, and news articles) that triggers negative affect	—^b^
Social comparison cues on social media that result in increased negative affect (DA group: 7/21 and comparison group: 0/7)	Social media content (eg, images, information on healthy lifestyle practices, and past positive life experiences) highlighting perceived personal faults Social media content (eg, images) highlighting opportunities that are no longer available to one’s self but to others who are similar Instructive content, especially user-generated, that is related to sensitive topics (eg, child-rearing) and that is contrary to personal practices	—
Abusive content limits Web usage by those who avoid it (DA group: 5/21 and comparison group: 0/7)	Avoidance of unfamiliar and news-related websites because of the fear of unintentionally accessing personally upsetting and inappropriate content Avoidance of social media participation because of the fear of receiving abuse	—

^a^DA: depression and anxiety.

^b^Not applicable.

#### Social Comparison Cues on Social Media That Result in Increased Negative Affect

Some content was effective at directing attention toward drawing upward comparisons between several participants in the depression and anxiety group and others, and participants in the depression and anxiety group and themselves in past. These comparisons were often negative and considerably upsetting:

I had one that came up this week that was a photograph of me, many years ago. Friend’s wedding. I was a bridesmaid. I just looked at this photograph and went, I mean, I looked good…I immediately felt that I’d let myself down. I thought, “Well look, clearly you can manage this. What’s happened?”

Clara, depression and anxiety group

No participant in the comparison group reported a similar difficulty.

#### Abusive Content Limits Web Usage by Those Who Avoid It

Some participants also refrained from commenting, posting updates, and engaging in various Web-based activities in fear of suffering abuse from other Web users as a result:

I left a comment, and then I just had a stream of abuse from people, because I voted to leave... The Web, in general, is quite a hostile place, and I don’t want to be in that sort of environment. It doesn’t make me feel particularly safe or comfortable, being online. As I said, I stick very much to what I know, because I feel quite unsafe outside of that.

Jason, depression and anxiety group

No participant in the comparison group reported a similar difficulty.

#### Theme 2: Lack of Safety, Privacy, and Security Controls

A total of 4 subthemes were identified within this theme: (1) lack of control over access and usage, (2) lack of safety controls, (3) threats to privacy, and (4) ambivalent contact. Ensuring safety for oneself and significant others when using the Web was described as a difficult task only by participants in the depression and anxiety group, as shown in Table 4. Similarly, except for 1 participant from the comparison group, difficulties pertaining to contact were reported by several participants in the depression and anxiety group (10/21).

#### Lack of Control Over Access and Usage

Table 4 shows several recreational activities on the Web that several participants (19/21) from the depression and anxiety group said displaced important tasks. Participants were unable to stop engaging in these activities even when they wanted to stop. Some participants said that these activities were an outlet for coping with unpleasant feelings and procrastination:

I discovered YouTube over the winter exams…When I’m overloaded in other areas, it’s like a release…I know I’m doing it. That does not mean I can stop.

Sara, depression and anxiety group

A high-level of ease of use was also attributed to making unintended purchasing, banking, and time management decisions, without giving due consideration. These features were considered as being too easy not to use:

There’s the one-click, it’s so easy just to go through and buy and buy and buy, and buy loads of stuff that you can’t really afford…Like I said, I have a tendency, sometimes, to make impulse purchases, and I’ll look and think I’ve got more money than I have, and before I know it I’m at the bottom of my overdraft again.

Jason, depression and anxiety group

#### Lack of Safety Controls

Some participants discussed how they grappled with complex issues relating to the differences in privacy approaches between countries and companies and the repercussions for what they self-disclosed to websites based on these factors. Other participants were concerned about keeping their children safe but admitted that they were unable to remain motivated to keep abreast of the constant changes in how safety was managed and circumvented on various websites. Several participants expressed their interest in realizing the wider benefits of the Web. However, they were forced to strictly limit their use of many websites, such as social media websites, and others narrowed their use of the Web to a limited number of websites in fear of abuse and receiving unsolicited contact.

The fear of being a victim of crime and getting involved in a conflict on the Web is equally as concerning as avoiding abuse and unsolicited contact. The result of dealing with this fear is often also limiting Web use. A lack of forewarning about the known types of service misuse, information on how to avoid safety pitfalls associated with the usage of Web-based services and a lack of support options in the event the user is negatively affected also presented difficulties for depression and anxiety group.

As demonstrated with theme 1, it is important that participants have a choice in what content they are exposed to, especially on social media platforms, as the emotional consequences can be profound. Participants were exposed to sensitive content regularly and were unable to avoid it effectively. The highly varied nature of sensitive content on the Web and the lack of control over exposure to it were the main reasons given for why this occurred:

People post videos of the dogs being boiled alive to raise awareness...It’s a really upsetting video, you don’t have the possibility to not want to play it, you got auto play on and you scroll through it, it would just start playing.

Jade, depression and anxiety group

No participant in the comparison group reported a similar difficulty.

#### Threats to Privacy

Participants in both groups were generally concerned about the privacy of their personal data. Participants in the depression and anxiety group identified many instances of where they particularly felt vulnerable, as shown in Table 5.

However, these participants sometimes also failed to take necessary precautions because of their felt sense of personal insignificance:

In terms of difficulties, it’s really kind of finding minor details for terms and conditions for various services and various things that you use online, whether it’s the rights that a social media platform has for your data or the rights of a purchasing website to then use your details in marketing. It’s very buried, I find.

Betty, depression and anxiety group

#### Ambivalent Contact

Several participants (10/21) in the depression and anxiety group experienced much difficulty with direct contact from social connections and stopping consistent contact from unknown senders:

My partner almost caused me to lose my life…I don’t follow him, I’m not friends with him. And then suddenly, about two weeks ago. On the bottom of photo, he wrote something…That’s really unsettling.

Hera, depression and anxiety group

If somebody messaged me personally I would always respond...I think it gets worse when I’m low...I find social interactions quite draining, when I’m already tired, because you kind of in a way have to put up a bit of a façade, which is obviously very hard to maintain.

Paisley, depression and anxiety group

Moreover, 1 out of 7 participants in the comparison group identified avoiding spam via social media platforms as being a difficult task.

**Table 4:** Difficulties because of a lack of safety, privacy, and security controls experienced by participants in both sample groups by subtheme.

Subtheme—number of participants from DA^a^ (n=21) and comparison (n=7) groups	Difficulty reported by the DA group participants	Difficulty reported by the comparison group participants
Lack of control over access and usage (DA group: 19/21 and comparison group: 1/7)	Addictively accessing similarly upsetting content (eg, news articles on similar topics) that is readily available Repeatedly clicking on posts and performing other actions on social media, news, and shopping websites Addictively performing online tasks that displace other tasks—gaming, gambling, and watching videos Keeping track of time on social media is difficult Coping with anxiety by fixating on finding answers to a salient issue online and avoiding activities (eg, accessing bank account in anticipation of a low balance) Easy achievable compulsion to set up bank overdrafts, make online purchases, and donate to charities Avoid online civic engagement because of a sense of insignificance Personal online shopping results in feelings of guilt Reluctantly using online dating when feeling low to increase feelings of self-worth	Addictively accessing social networking services and news websites

Lack of safety controls (DA group: 11/21 and comparison group: 0/7)	Understanding how to protect one’s family from online dangers and being confident enough to do so Unable to anticipate if a news article will be upsetting Detecting scams and phishing attempts on banking platforms Limiting Web usage by only using familiar websites to avoid unknowingly committing criminal acts Lack of control over exposure to content Trusted websites that occasionally feature links to unsafe websites	—^b^
Threats to privacy (DA group: 10/21 and comparison group: 1/7)	Fear that data from personal data breach would be sold to third parties, or fear of being hacked A sense of insignificance discourages the implementation of privacy measures on social media platforms Frustrating when personal data sharing, including seemingly unnecessary personal data, is required to participate in online activities Targeted advertising using posts, especially posts shared during a depressive episode Distressing having comments publicly visible Finding and understanding terms and conditions policies and keeping abreast of changes	Ensuring privacy and safety online—identifying scams and scammers
Ambivalent contact (DA group: 10/21 and comparison group: 1/7)	Fear of direct contact or contact beyond a “*like*” or similar form of engagement, from social connections, especially during a depressive episode Uncertainty about how to stop contact—being removed from electronic mailing lists Mandatory contact to obtain resources—subscription to electronic mailing lists Making contact—connecting with people through video clips and reading news instead of direct contact, avoid responding to messages as it is mentally effortful, and pressured to respond to messages immediately	Avoiding spam via online social networking sites

^a^DA: depression and anxiety.

^b^Not applicable.

**Table 5:** Difficulties because of having a lack of adequate support experienced by participants in both sample groups by subtheme.

Subtheme—number of participants from DA^a^ (n=21) and comparison (n=7) groups	Difficulty reported by the DA group participants	Difficulty reported by the comparison group participants
Lack of support for error recovery and overcoming emotional difficulties (DA group: 17/21 and comparison group: 3/7)	Managing subscriptions is frustrating—lack of forewarning, automatic renewals, and difficulty requesting and obtaining refunds Remembering many passwords and special codes Lack of clear warnings about the risks associated with online dating on particular platforms and of support when things go wrong Posting content is mentally effortful and time-consuming—choosing emoji, expressing feelings without causing alarm, and fear of using incorrect grammar Immediately quit or desperately and hastily try many strategies to complete challenging tasks Having too many options and information results in indecisiveness and distraction Impersonal social messaging feature makes taking the first step to seek help from connections difficult Feeling ignored when social connections do not react to personal posts Sharing content is a struggle—highlighting personal positives, fear of attracting abuse and attention from others, making offense or causing conflict, and fear of sharing inaccurate and uninteresting content Difficulty learning from onscreen material because of an inability to engage actively Difficulty getting support online, given the inflexibility of online banking and education support systems that are often not user-friendly Unable to accurately gauge reactions when interacting with others via online dating platforms Fear of opening messages and being pressured to respond to a message immediately as a read receipt has been sent	Fast time-outs
Information gathering on the Web (DA group: 12/21 and comparison group: 3/7)	Choosing the right search keywords for difficult-to-find resources Time-consuming to assess the veracity of information on the Web Hard to keep focus and understand information online during a depressive episode Getting distracted when navigating across many websites to find needed information Search results listing multiple sources with identical information and few sources with original content Assessing the availability of resources across multiple academic databases Not knowing when to stop searching for and evaluating online resources Quit searching the Web in frustration after not finding needed results Processing too much information from many sources makes it difficult to ascertain if one’s search is complete Easily distracted when browsing the Web during a depressive episode Sites that break up content across pages to increase advert views	Selecting the effective Web search terms Missing information—important information that should be online but is not Reading multipage articles

^a^DA: depression and anxiety.

#### Theme 3: Lack of Adequate Support

A total of 2 subthemes were identified within this theme: (1) lack of support for error recovery and overcoming emotional difficulties and (2) information gathering on the Web. The majority of participants (17/21) in the depression and anxiety group and 3 out of 7 participants in the comparison group highlighted that a lack of meaningful support made using the Web challenging (Table 5). Difficulties with information gathering online were also identified by 12 out of 21 participants in the depression and anxiety group and 1 out of 7 participants in the comparison group.

#### Lack of Support for Error Recovery and Overcoming Emotional Difficulties

Several participants in the depression and anxiety group believed that they were not given the necessary support by websites to overcome difficulties, especially when they were feeling unwell:

I make lots of mistakes don’t get me wrong. I got delivered five kilos of bananas the other day...They could have a “do you need” button before you submit…“Are you sure you need five kilos or five bananas?”

Christine, depression and anxiety group

They also pointed out that websites often compounded this situation by not automatically correcting obvious user errors and, instead, sometimes made completing tasks more effortful as a result. Existing support options were not helpful as they were often not user-friendly and did not address difficulties common to participants. These participants shared that remaining motivated to solve the reported difficulties independently of others was challenging:

It depends on how tired I am. If I can’t get what I want immediately I give up. Then I’ll shelve it, and I’ll come back. If I need it really urgently, then I just try lots of different things.

Hera, depression and anxiety group

Several participants (3/7) in the comparison group shared difficult experiences with form timeouts that terminated too quickly.

#### Information Gathering on the Web

Gathering information using the Web, especially via search engines and reading multipage articles, proved challenging for participants in both groups (Table 5):

Things that I find difficult are getting that...putting the right stuff into your search...So that you get the information you want, and when you know something’s out there, but you can’t easily get to it.

Hera, depression and anxiety group

Then there’s one picture on one page then you have to scroll to another page to the next part of the article...I find that really frustrating.

Christine, depression and anxiety group

Participants (12/21) in the depression and anxiety group experienced additional difficulties in remaining focused when searching the Web using databases and browsing across multiple websites (Table 5):

Where you have to click to go to the next. You know they’re just doing that, I feel, to measure their clicks so they know how far you’re getting in the story, is what I feel. Especially if I’m just doing it on my phone. I have a cheaper phone. It’s not so fast. I think, “Oh okay, forget it.” This is annoying when you could just put the content right there one page.

Kurt, depression and anxiety group

#### Theme 4: Difficult User Interfaces

A total of 2 subthemes were identified within this theme: (1) using complicated and effortful user interfaces on the Web and (2) malfunctioning websites. Participants in both groups recounted frustrations using complicated and malfunctioning websites (Table 6).

#### Using Complicated and Effortful User Interfaces on the Web

Unintuitive websites presented difficulties for participants in both groups (Table 6). Participants (14/21) in the depression and anxiety group recalled experiences involving taking regular breaks and frustratingly struggling until they were able to complete tasks such as reading and shopping online:

Why do we have to have pop-ups? It kind of perplexes me why it’s so invasive. You just kind of feel like...You almost want to flip channels but you can’t.

Betty, depression and anxiety group

You can sit down with a fixed idea of what you would like and then by looking on the web you’ve got so many different products...You then pull back from the decision because there’s too much to decide from.

Jason, depression and anxiety group

Participants in the comparison group (3/7) discussed difficulties completing long website forms and constantly changing user interfaces for frequently used services.

#### Malfunctioning Websites

Participants in both groups identified difficulties with unresponsive websites, feedback, and page loading errors (Table 6). Experiences with malfunctioning websites sometimes led to catastrophic thinking and a reduced willingness to troubleshoot by those in the depression and anxiety group:

I tend to try and avoid going onto my app or looking at my bank statement as much as possible because it makes me really worried. I’ve actually seen it takes twice as long to log you in so it’s almost like the wait and the panic that what little money you’ve got is taking longer.

Trish, depression and anxiety group

**Table 6:** Difficulties with challenging user interfaces experienced by participants in both sample groups by subtheme.

Subtheme—number of participants from DA^a^ (n=21) and comparison (n=7) groups	Difficulty reported by the DA group participants	Difficulty reported by the comparison group participants
Using complicated and effortful user interfaces on the Web (DA group: 14/21 and comparison group: 3/7)	Unintuitive user interfaces for education on the Web—time-consuming to find course materials, complicated academic databases, and difficult reading via Web-based reading services Unintuitive user interfaces for banking on the Web—intimidated by terminology and abundance of numbers, unclear system feedback, and setting up new bank recipient is complicated Relearning user interfaces after changes is difficult, especially when lacking the motivation to explore	Completing long website forms Constant user interface changes
Malfunctioning websites (DA group: 11/21 and comparison group: 2/7)	Sites not optimized for mobile browsing and poor connectivity Malfunctioning critical website feature delaying completion of an important task Frustrating to be given options that are not available Bad video streaming experiences because of poor connectivity Catastrophic thinking because of malfunction or irregularities in the operation of the website Remaining motivated to independently resolve complicated problems caused by websites	Unresponsive websites Page loading errors

^a^DA: depression and anxiety.

^b^Not applicable.

#### Summary: The Most Common Difficulties People With Depression and Anxiety Encounter When Using the Web

A total of 8 subthemes describe difficulties experienced by more than half of the participants in the depression and anxiety group (Table 7).

#### Web Activities, Services, and Features for Which the Highest Number of Difficulties Were Reported

Web activities, services, and features being used by participants when they experienced difficulties were also reported (Table 8). Table 8 shows 19 of these for which a higher number of difficulties were reported than the average number of difficulties reported for a Web activity, service, or feature.

**Table 7:** Most common difficulties by subthemes with number of participants in both groups affected.

Difficulty subtheme	Participants in the people with depression and anxiety group affected (n=21), n	Participants in the comparison group affected (n=7), n
Lack of control over access and usage	19	1
Unexpected and irrelevant content is upsetting	17	6
Lack of support for error recovery and overcoming emotional difficulties	17	1
Features and content that are reminders of upsetting experiences and negative affect triggers	14	0
Understanding complicated user interfaces on the Web	14	2
Information gathering on the Web	12	2
Malfunctioning websites	11	2
Lack of security controls	11	0
Privacy risks	10	1
Ambivalent contact	10	1
Social comparison cues on social media that result in increased negative affect	7	0
Abusive content limits Web usage by those who avoid it	5	0

**Table 8:** Web activities, services, and features for which the highest number of difficulties were reported.

Web activities, services, and features	Difficulties reported from the people with depression and anxiety group (n=167), n	Difficulties reported from the comparison group (n=16), n
Facebook	56	3
General Web usage	22	2
News sites	19	0
Adverts	17	0
Online learning	17	0
Online banking	16	2
Online shopping	16	1
Conducting research online	13	0
Content sharing by connections on social networking services	11	1
Posting content	10	0
Business-related Web usage	9	0
Online search	11	0
Online dating	8	0
YouTube	8	0
Twitter	7	1
Online civic engagement	6	0
Commenting feature	6	0
eBay	5	0
Instagram	5	0

### Study 2—A Mental Health Practitioner Expert Web-Based Survey Study

Data were collected from 21 respondents (4 males and 17 females) aged between 30 and 72 years using purposive sampling (Table 9).

MHPs reported 10 perceived difficulties relating to Web usage by the people with depression and anxiety. Of these, 2 difficulties were only relevant to personas diagnosed with depression. The remaining 8 difficulties were shared by personas diagnosed with either depression or an anxiety disorder. The 10 perceived difficulties were linked to 22 common impairments, limitations in activities of daily life, and diagnostic criteria associated with depression and anxiety. These difficulties are organized under 3 themes: (1) navigating and generally operating websites, (2) content on the Web, and (3) lack of trust in the Web.

#### Theme 1: Navigating and Generally Operating Websites

MHPs (n=19) identified 5 perceived difficulties within this theme that focus on the general usage of websites by people with depression and anxiety (Table 10). All 4 personas are captured in this theme.

#### Navigating the Web

Using the Web was generally seen by experts (n=10) as involving many effortful activities that could pose challenges for how people with depression and anxiety perceived, understood, and used Web-based resources. For example, Web browsing was often pinpointed as a potentially difficult task. Personas with either condition were thought to be lacking the necessary motivation and energy to effectively use Web-based resources and the ability to solve emergent problems. Experts believed that these difficulties could be compounded by impaired emotion regulation, poor concentration, and the physical manifestations of their conditions, for instance, an upset or worried user experiencing difficulty navigating a website along with finding it hard to concentrate on the task at hand.

#### Malfunctioning and Unintuitive Sites

Malfunctioning websites and websites with an unintuitive design were also thought by experts (n=7) to be especially difficult for people with depression and anxiety to use. These websites were described as using too small font sizes, unnecessarily bright colors, and many shapes within its design. The experts expressed concern that members of this group were often fatigued and might also struggle with remaining resilient when encountering these experiences. Feelings of hopelessness, worthlessness, and worry were mentioned as possible outcomes.

**Table 9:** Sample demographics of mental health practitioner expert Web-based survey study.

Characteristics			Mental health practitioner experts (n=21), n
**Sex**			
	Male		4
	Female		17
**Age** **(years)**			
	30-49		2
	50-69		16
	≥70		3
**Years of experience**			
		5-10	3
		11-15	5
		>15	13
**Profession**			
	Counselor		10
	Clinical psychologist		1
	Psychiatrist		1
	Psychotherapist		8
	Occupational psychologist		1
**Country**			
	United Kingdom		20
	Ireland		1

**Table 10:** Perceived difficulties navigating and generally operating websites.

Perceived difficulties	Associated impairments, limitations in activities of daily life, and diagnostic criteria
Navigating the Web^a,b^	Lack of motivation, lack of energy, impaired emotion regulation, poor concentration, physical symptoms (eg, tingling or numb fingers, dizziness, and shortness of breath), and difficulty solving problems
Malfunctioning and unintuitive sites^a,b^	Poor concentration, lack of motivation, low resilience, worry, low mood, low self-confidence, low self-esteem, fatigue, feelings of hopelessness and feelings of worthlessness
Effortful tasks^a,b^	Lack of motivation, worry, impaired emotion regulation, poor concentration and feelings of hopelessness
No clear guidance on how to complete tasks^a^	Poor concentration and feelings of hopelessness
Excessively detailed websites with information/design elements^a,b^	Feelings of being overwhelmed and lack of energy

^a^Difficulty reported for persona with depression.

^b^Difficulty reported for persona with an anxiety disorder.

#### Effortful Tasks

Several common online activities were highlighted by experts (n=7) as possible areas of difficulty because of the sustained mental effort involved. These activities included seeking help online for technical and personal issues, reading, completing forms, and conducting online research. Experts noted that feelings of hopelessness and worry coupled with a lack of motivation and poor concentration might make these activities challenging for those affected by depression and anxiety.

#### No Clear Guidance on How to Complete Tasks

The potential for a Web activity to pose difficulty was thought to be increased when no clear guidance on how to complete the necessary tasks was provided. Experts (n=4) believe that this fosters a feeling of hopelessness within users with depression and anxiety.

#### Excessively Detailed Websites With Information/Design Elements

Some experts (n=3) also characterized websites featuring excessive amounts of information and design elements as being barriers to effective use. It was feared that information overload would be the likely result and that it would overwhelm people with depression and anxiety who tend to be already low on energy.

#### Theme 2: Content on the Web

MHPs (n=10) identified 3 perceived difficulties within this theme relating to the perception and comprehension of website content by people with depression and anxiety (Table 11). All 4 personas are captured in this theme.

**Table 11:** Perceived difficulties with content on the Web.

Perceived difficulties	Associated impairments, limitations in activities of daily life, and diagnostic criteria
Retaining information^a,b^	Poor concentration, worry, fatigue, low self-confidence, and physical symptoms (eg, tingling/numb fingers, dizziness, and shortness of breath)
Content that does not resonate^a,b^	Negativity bias, lack of motivation, impaired emotion regulation, low self-esteem, feelings of being overwhelmed, and feelings of isolation
Content that triggers repetitive thinking^a,b^	Impaired emotion regulation

^a^Difficulty reported for persona with depression.

^b^Difficulty reported for persona with an anxiety disorder.

#### Retaining Information

The experts (n=4) shared that retaining information on websites would be difficult for users with these conditions. Symptoms including poor concentration, worry, fatigue, and physical symptoms such dizziness were cited as factors that contribute to this outcome.

#### Content That Does Not Resonate

Experts (n=9) believed that online content lacking personal meaning or importance to users and content that users perceived as overly positive or negative would present several challenges for this group. Experts stated that this kind of unbalanced content could be demotivating, overwhelming, and isolating for this group. Overly negative content was believed to have the added potential to affirm negative fears and concerns. This difficulty was noted for all personas as well:

He may have difficulty feeling the wording on a website applies to him if he does not feel directly spoken to in an understanding way by what is written on a website.

MHP 1

#### Content That Triggers Repetitive Thinking

Content that is reminiscent of negative personal experiences was highlighted as a potential challenge by an expert (n=1) for 2 of the personas. Words or phrases that might be associated with these experiences were deemed to have the potential to easily take users on a negative mental tangent where they would repeatedly focus and refocus on negative personal experiences. This kind of repetitive negative thinking [39,40] is believed to present difficulties for concentration and the comprehension of online content as well.

#### Theme 3: Lack of Trust in the Web

MHPs (n=4) identified 2 perceived difficulties within this theme relating to information sharing by users and their safety online (Table 12). A total of 3 personas—2 diagnosed with depression and 1 an anxiety disorder—are captured in this theme.

**Table 12:** Perceived difficulties relating to a lack of trust in the Web.

Perceived difficulties	Associated impairments, limitations in activities of daily life, and diagnostic criteria
Self-disclosure online^a,b^	Worry, perfectionism, and low self-confidence
Feeling safe online^a^	Worry, feelings of vulnerability, and withdrawal

^a^Difficulty reported for persona with depression.

^b^Difficulty reported for persona with an anxiety disorder.

#### Self-Disclosure Online

Sharing personal information online was highlighted as a potential challenge for people in this group. It was mentioned that users might experience great worry about how their information might be used beyond what was intended. Sharing personal information in what seems to be a public setting may also be difficult for users who are experiencing low self-confidence issues. It was also mentioned that some users might worry about making mistakes and become overly concerned about completing information collection forms correctly.

#### Feeling Safe Online

Ensuring one’s safety was also identified as a potential difficulty for people with depression. Fear of privacy violations, exploitation, deception, and crime were some of the issues highlighted. Experts say these fears foster intense feelings of worry and vulnerability that can be mentally debilitating and are believed to result in users withdrawing from some aspects of the Web.

## Discussion

### Principal Findings and Comparison With Prior Work

This investigation identified and described many of the difficulties people with depression and anxiety experience on the Web through triangulation using 2 data sources. First, study 1 benefited from face-to-face interviews with comparison group participants that were used to highlight unique difficulties identified in face-to-face interviews with participants from the depression and anxiety group. Second, study 2, a persona-based Web-based expert survey, involved MHPs who identified many difficulties that were also reported by the people with depression and anxiety in study 1 and further described these difficulties from a clinical perspective. Findings from this investigation have contributed to a more robust and fuller understanding of the difficulties people with depression and anxiety experience online and provide actionable insight to researchers, engineers, policy makers, and clinicians in their practice.

Participants in the depression and anxiety group reported a higher number of the identified difficulties compared with those in the comparison group. Furthermore, difficulties reported by participants in the depression and anxiety group were discussed within the context of a larger number of Web activities, services, and features when contrasted with the difficulties reported by the participants in the comparison group. In line with research on resilience [41,42], difficulties reported by participants in the depression and anxiety group were more detailed and considered to be more detrimental by those in this group when contrasted with those reported by participants in the comparison group.

Perceived difficulties identified by MHPs in study 2 showed considerable overlap with those identified in study 1 by participants in depression and anxiety group. Nonetheless, the investigation benefited a great deal from using triangulation. People with depression and anxiety clearly detailed the difficulties they experienced, at times also mentioning the harmful consequences, and MHPs suggested linkages between difficulties reported by the people with depression and anxiety and the common impairments, limitations in activities of daily life, and diagnostic criteria associated with their conditions.

Inappropriate and personally sensitive content was instrumental in triggering persistent and cyclic negative thinking that is characteristic of rumination in depression and anxiety [43] and was linked to impaired emotion regulation by MHPs in study 2. This content was also found to encourage negative social comparisons that exacerbated anxiety symptoms and negatively affected daily functioning [44]. Researchers [45] have found that the more Web users with the inclination toward social comparisons engage in this behavior online, the more they experience negative feelings. Findings from some studies [46] also show that exposure to content that supports clear social comparisons is more detrimental to women with a high tendency to compare themselves with others, relative to women without this tendency.

MHPs in study 2 tied the concern of people with depression and anxiety for safety and privacy controls on the Web to a general lack of trust in the Web. Similar to this investigation’s findings, research [47] has associated psychological distress with frequent unwanted contact or communications overload because of the usage of multiple online communication channels. Receiving unwanted contact and misunderstandings were identified as the most common negative Facebook experiences reported by young adults in another study [48] and were associated with depressive symptoms as well. The fear of receiving negative feedback online and coping with the pressure to maintain social network updates has also been reported in other studies [49,50]. Moreover, research [51] has also revealed that social anxiety is positively related to a concern for privacy on the Web and that this concern is negatively related to self-disclosure online as well. Safety issues on the Web for people with depression and anxiety also involve distracting features of social media that facilitate procrastination and are likely being used to avoid stressful but necessary tasks [52]. Andreassen and Pallesen [53] found that investing too much time and effort into using social media can negatively impact other social activities, relationships, and well-being. Moreover, despite not being identified by MHP, people with depression and anxiety in other studies [54,55] also shared experiences that involved compulsive buying behavior.

People with depression and anxiety identified many features—regarding making subscriptions easier to manage, issuing notices about known risks on platforms, ability to easily reduce options to choose from, and the ability to have more flexible service support options—that could be implemented to help them overcome many of the difficulties they face when using the Web. Although MHPs did not identify the majority of the difficulties in this theme, these practitioners highlighted difficulties people with depression and anxiety might experience with understanding excessively detailed websites [56] and navigating the Web, which were also mentioned by the people with depression and anxiety.

Both people with depression and anxiety and MHPs identified malfunctioning, unintuitive, and effortful to use sites as descriptions of websites that may pose difficulties to people with depression and anxiety. MHPs suggest the interaction between several characteristics of depression and anxiety (eg, fatigue, poor concentration, and lack of motivation) [9,36] and the aforementioned features of the Web as the reason for the resulting difficulties as described in this paper.

The most common difficulties experienced by participants with depression and anxiety in study 1 were encountered when using the most common Web activities, services, and features mentioned when talking about difficulties. For example, unexpected content is especially common on social networking platforms, news sites, and in online advertisements. Given that the sample had a majority of young participants, the most common Web activities, services, and features where they experienced difficulties were somewhat expected.

### Limitations

Though the study benefited from having a mostly young and predominantly female sample in several ways, this may have limited the range of Web activities the sample engaged in and, therefore, also the range of difficulties identified. Similarly, the difference in sample size between the participant groups in study 1 may have also limited the range of difficulties identified for the smaller comparison group. However, data saturation determined the final number of participants for each group, and therefore, no new information that would have enhanced or changed the findings of a study was expected. Nevertheless, this is one of the first studies of its kind, and this population can serve as a good example for future studies with more diverse and larger samples. Moreover, as the small sample sizes in study 1 limit the ability of the independent samples *t* test to detect a statistical difference in Web skills between the depression and anxiety and comparison groups, the results of this analysis should be cautiously interpreted.

Although unlikely, participants in the comparison group could have misreported a past mental disorder diagnosis. However, these participants were also screened using the Patient Health Questionnaire for Depression and Anxiety, and all of them had a score between 0 and 2 at the time of study.

Despite piloting, some MHPs in study 2 deemed the survey as being too long. This sometimes resulted in receiving a few repetitive responses and complaints about the amount of effort necessary to complete the survey. Conducting screening that considers the necessary levels of attentiveness and effort that are needed for such surveys is of utmost importance for future studies involving this group.

Given these limitations, it is important to note that using data triangulation would have also helped reduce the negative impact of these limitations and improved the robustness of this study’s findings as well.

### Implications and Recommendations for Practice and Future Research

Findings of this investigation are accessible to researchers in different disciplines to build on, engineers working on the development of accommodating Web-based resources, clinicians who need to be informed about the challenges their patients face in everyday life, and policy makers who can create evidence-based policies that can together realize very positive outcomes for people with depression and anxiety. These findings also place the spotlight on the importance of considering difficulties associated with affective states when delivering enablement initiatives involving the use of technology. This is in contrast to the substantial degree of attention given to the needs of those with sensory impairments and physical disabilities. Researchers are also encouraged to adopt a more comprehensive view of accessibility that captures the complexity of the interaction between users and their environment.

The International Classification of Functioning, Disability, and Health’s Model of Functioning and Disability provides a clear framework that can be used to describe and study the user experience of people with mental health conditions when using Web-based and other digital technologies. For example, the World Wide Web Consortium’s Four Principles of Accessibility [57] focuses on instances where a Web-based resource is not understandable, perceivable, and operable but neglects the other important factors in the interaction between the user and the user’s environment.

The high variability in user needs among people with depression and anxiety presents a unique challenge for accessibility professionals. Enablement efforts should be targeted at an individual level and no longer solely at a user group level. Meeting this challenge will call for new facilitation methods that rely on emerging technologies such as artificial intelligence to provide highly personalized experiences for each user with depression and anxiety and also other mental health conditions.

The adoption of stronger data protection policies (eg, [58]) will be of great benefit to people with depression and anxiety who worry about not having enough control over their personal data and having it be misused for privacy violations (eg, fear of unwanted direct contact) among other infractions.

### Conclusions

People with depression and anxiety experience difficulties when using the Web that are related to sociocognitive deficits associated with their conditions. Participants in the comparison group did not experience most of these difficulties. MHPs are very aware of the difficulties that people with depression and anxiety are likely to experience when using the Web. Findings highlight several Web activities, services, and features that should be reviewed not only for people with depression and anxiety but also for people affected by other mental disorders and conditions that share similar symptomology. This investigation has contributed to a fuller understanding of these difficulties and provides guidance on what to address for people with depression and anxiety when using the Web. It also calls for novel approaches to aid in the removal and reduction of these difficulties using more carefully personalized experiences.

## Appendix

Multimedia Appendix 1 Semistructured interview topic guide.

Multimedia Appendix 2 Checklist for Reporting Results of Internet E-Surveys.

Multimedia Appendix 3 Mental health practitioner expert Web-based survey questions.

## Abbreviations

MHP: mental health practitioner
DA: depression and anxiety
