# Supplementary material for: Difficulties Encountered by People With Depression and Anxiety on the Web: Qualitative Study and Web-Based Expert Survey
Source: J Med Internet Res. 2019 Oct 31;21(10):e12514. doi: 10.2196/12514 (PMC6914249; doi:10.2196/12514)
Supplement: Multimedia Appendix 1 [file jmir_v21i10e12514_app1.pdf]

Multimedia Appendix 1. Semi-structured interview topic guide.

Ice Breakers

- What thoughts come to mind when you think about the Web?
- What places do you access the Web from?
- How do you normally access the Web? For example, via your mobile, laptop, work computer, tablet.
- How do you spend your time online?

Difficulties Experienced When Using the Web

So you've indicated on the screening form that you use the Web for several activities: social networking, online dating, job search, gaming, movies, music, books, shopping, online banking, eLearning and education, online civic engagement, information gathering. I would like to now go through these activities.

- How often do you use the Web for ________?
- Can you tell me a bit about when you use the Web for this activity?
- Could you describe any positive and or negative experiences you may have with this activity? *[Probe: What do you have trouble with? What tasks often lead to mistakes? What tasks do you avoid? What tasks would you like help with when using the Web?]*
- Have you noticed any changes in your experience of these difficulties? *[Probe: Are they getting worse, better, going away or returning?]*
- If it was up to you, what would you change to help you remove or reduce this difficulty?

Privacy, Cyber-security and safety

- What are your thoughts on online privacy, security and safety?
- What steps have you taken to ensure your security and safety online?
