# Supplementary material for: Difficulties Encountered by People With Depression and Anxiety on the Web: Qualitative Study and Web-Based Expert Survey
Source: J Med Internet Res. 2019 Oct 31;21(10):e12514. doi: 10.2196/12514 (PMC6914249; doi:10.2196/12514)
Supplement: Multimedia Appendix 2 [file jmir_v21i10e12514_app2.pdf]

Multimedia Appendix 2. Checklist for reporting results of Internet e-surveys (CHERRIES).

Table 1. Checklist for Reporting Results of Internet E-Surveys (CHERRIES) - Study 2.

Item Category	Checklist Item	Explanation
Design	Describe survey design	Purposive sampling was used to recruit participants for this survey. Respondents had to be aged 18 or above, a mental health professional who is an accredited, chartered or registered member of a professional body and currently treats people with depression or an anxiety disorder. Data saturation determined the final number of respondents.
IRB (Institutional Review Board) approval and informed consent process	IRB approval	Ethical approval was granted by the ethics committee of the University of Southampton.
	Informed consent	Potential participants were emailed the participation information sheet (PIS) — study description, reasons for contact, description of participation, benefits of participating, likely risks, terms of confidentiality, options for further information — and asked to consider participating for at least 24 hours before making a decision. After this time they were permitted to use a link in the email to the Web-based informed consent form — indicated understanding of PIS and opportunities to obtain further information and voluntary nature of participation, agreement to participate —, which was activated 24 hours after the email was sent. Only those giving consent were allowed to progress to participate in the Web-based survey.
	Data protection	The study will be compliant with the Data Protection policy of the School of

		Psychology, University of Southampton and the Data Protection Act (1998). All data will be stored on a password-protected device in encrypted format. No hard copies of survey responses will be stored.
Development and pre-testing	Development and testing	The survey asked demographic questions (e.g., education background and expertise) and open-end questions about the personas that were provided (see Multimedia Appendix 3). The 4 personas used were fictional characters (2 with depression and 2 with anxiety) developed for the present study by RB based on information about impairments, activities limitations and participation restrictions experienced by persons with depression or anxiety, diagnostic criteria associated with these conditions, and also scenarios that feature a wide range of common Web activities. Feedback on the first version of the survey was obtained from two mental health professionals who participated in the survey pilot and some modifications were made as a result.
Recruitment process and description of the sample having access to the questionnaire	Open survey versus closed survey	The survey was essentially an “open survey” but was only accessible by those with a special link.
	Contact mode	Respondents were recruited from an online database directory of mental health professionals who are accredited, chartered or registered members of a professional body in the United Kingdom. Potential participants were contacted by the managers of the online database directory via email.
	Advertising the survey	Potential participants were contacted by the managers of the online database directory via email.

Survey administration	Web/E-mail	A website was used.
	Context	The survey was built and hosted using an online survey platform.
	Mandatory/voluntary	Participation was voluntary.
	Incentives	Participants were notified that there were no direct benefits associated with their participation. They were informed that they would be making a crucial contribution to the field of Web accessibility where the Web can be further developed to better accommodate people with depression and anxiety.
	Time/Date	The survey ran between January and October 2016.
	Randomization of items or questionnaires	Participants answered questions relating to 2 of the 4 personas that were randomly given. One persona focused on depression and the other on an anxiety disorder.
	Adaptive questioning	Not applicable.
	Number of Items	19 items.
	Number of screens (pages)	3 pages (7, 6 and 6 items respectively)
	Completeness check	All survey items were deemed to be mandatory, and respondents prompted to complete outstanding items before leaving the survey page on which the item was contained.
	Review step	Respondents were able to review and change their answers by navigating to previous and next pages.
Response rates	Unique site visitor	225 unique site visitors. A combination of IP address, location and cookies was used to determine unique visitors.
	View rate (Ratio unique site visitors/unique survey visitors)	Not applicable. Potential participants were invited through an external channel.
	Participation rate (Ratio unique survey page visitors/agreed to participate)	(42/225) 19%

	Completion rate (Ratio agreed to participate/finished survey)	(21/42) 50%
Preventing multiple entries from the same individual	Cookies used	Assigned on the first screen. Uncertain about how long the cookie was valid.
	IP Check	Used to ensure only unique participants completed the survey.
	Log file analysis	Not used.
	Registration	Not used but participants received a special link to gain access.
Analysis	Handling of incomplete questionnaires	Only completed questionnaires were included in the final dataset.
	Questionnaires submitted with an atypical timestamp	No respondents were removed from the survey for completing the items too quickly.
	Statistical correction	No methods such as weighting of items or propensity scores have been used to adjust for the non-representative sample.
