# Supplementary material for: Difficulties Encountered by People With Depression and Anxiety on the Web: Qualitative Study and Web-Based Expert Survey
Source: J Med Internet Res. 2019 Oct 31;21(10):e12514. doi: 10.2196/12514 (PMC6914249; doi:10.2196/12514)
Supplement: Multimedia Appendix 3 [file jmir_v21i10e12514_app3.pdf]

Multimedia Appendix 3. Mental health professionals expert Web-based survey.

Getting to know you

What is your age?
18-29
30-49
50-69
70 and over

What is your gender?
Male
Female
Other

How many years experience do you have in the field?
up to 5 years
5 to 10 years
10 to 15 years
over 15 years

What is your current job role?

Which country do you predominantly work in?

Personas

Vishal Mehta

I am 22 and graduated with an undergraduate degree in marketing last year after 3 years of studying in London. I fell in love with the city while studying and want to remain here for the foreseeable future to work or perhaps further my studies. I am originally from Mumbai where my family lives.

I was diagnosed with depression during my first year studying. I remember feeling lonely living in a new city far away from my family and eventually had little motivation for studying. I felt guilty for failing several classes in the first year and was fearful that I might not do well in the remainder of the degree. I had a really low mood during this time and little confidence in my ability to successfully continue my studies. I also lost interest in doing the things I normally enjoyed like football, playing the harmonica and writing.

My doctor assisted me in finding help. I mainly got support from my therapist and close friends from university who were aware of my condition. I did not discuss my condition with my family as I did not want them to worry. Despite feeling better now, I still struggle with depression and often have difficulties with concentration, making decisions and remembering important details.

I rely heavily on the Web for getting support from friends and family. I use Facebook and other online services to connect and stay in contact with friends, who mostly now live outside of London. Though my family is not aware of my condition, engaging and staying up-to-date with what's happening in their lives via their posts on these online services is important to me.

I have been actively looking for a job in London since graduating last year and have encountered several challenges. I often think I am not good at anything and had difficulty being motivated enough to adequately prepare application documents, update my CV and online professional profiles (e.g., LinkedIn), ask for recommendations and solve the problems that emerged along the way. I often feel tired and doing any task is an extra struggle. I haven't checked the status of the positions I finally applied for because I probably was not successful. Like who is going to select me? I am worthless. After a year of no success, I am now also thinking about going to graduate school. I begun to research graduate courses and funding opportunities online. I asked for assistance from others with this but found it difficult to discuss my challenges and to then act on the advice they gave. Needless to say, it hasn't been easy.

Julia Williams

A few years before starting college, when I was 19, I was diagnosed with depression. However, I soon after started therapy and found it helpful for managing my depression. I am now 45, married and have two young children. I have also been an elementary school teacher for the past 20 years since receiving my college degree in education.

Following the recent loss of my husband's job, my family and I moved from Toronto to the countryside. The countryside promises a more affordable lifestyle and better job opportunities for my husband who works in the leisure industry.

I began teaching at a local school soon after arriving and my husband meets potential employers on the outskirts of the local area often. Therefore, I am responsible for most household duties while actively seeking ways for the kids to adjust to this new lifestyle and integrate into our new community. However, the transition has not been easy for the family and I feel responsible for their difficulties adjusting.

I have been experiencing recurring health issues that forces me to take leave from work and also doing some of my chores at home. My doctor believes these issues are primarily linked to mental factors and that my depression is resurfacing. I told him about my difficulties managing my emotions and being overly preoccupied with my problems instead of finding solutions. My husband is supportive but he's often really not sure how to best lend support. Due to a lack of treatment options in my area, my doctor thinks I should consider Web-based options like online therapy and participating in online mental health communities.

The Web has been instrumental for me living in the city and it has become even more helpful since the move. I could research local activities for the kids, purchase necessities at cheaper prices online, manage the family's banking and financial obligations, and find resources for my classes. While doing these things online are usually easier, I've been struggling to make much effort to do the simplest of tasks recently. It's just been a difficult time.

Conny Armstrong

I was always a worrier. I would worry about my family and friends, health, schoolwork, everything from a young age. When worried, I often felt tense, my stomach would churn and I sometimes even broke out in a sweat and had palpitations. It didn't take much to scare me either and was almost always irritable.

Now at age 35 and a single mother of one, there is much more to worry about. My daughter will start primary school this year, I recently got a big promotion, and I'm afraid I will regret not making enough time for my daughter or myself. Therapy has been somewhat helpful over the years but I hardly have time for that nowadays, especially given my demanding job.

I'm a Web analytics manager at an agency in Boston. A great deal of my work focuses on analyzing customer online behavior on various types of websites. This insight is crucial for advising our clients on how successful their campaigns are and what can be done to make improvements.

I absolutely love my job and have worked hard for this promotion. However, I now worry a lot about how I am going to cope with this added responsibility. Sure it comes as no surprise that I'm losing much sleep thinking about it all and this in turn makes it all worse as well. I've been considering the idea of working remotely from home a couple days a week so I could be there when my daughter is home from school. Managing projects and the team remotely has its disadvantages though. My work though heavily Web-based is very hands-on and calls for much discussion at times. We would need to rely more on our online collaborative tools.

Adrian Eriksen

I recently moved to Grenada to work on an architectural project for 6 months. I've been struggling to get sleep at night since I arrived. I just lay there and a million thoughts bombard my mind for hours. On the flip side, I've also been regularly having trouble concentrating at work during the day. My mind often just goes completely blank. Reading a book or doing research online can be very difficult.

There are also those times recently I felt dizzy and my fingers went numb or got tingly. It all seems so unreal at times, especially when I feel like I can't breathe. I guess it's all due to my anxiety re-emerging as I recognize some experiences from having such problems in the past. However, some of these symptoms are new and scary to me. Before it was mostly a case where I always imagined situations being much worse than they were in reality. When I had a headache, I would think it is a

tumor. Now I guess it's much more physical, random and unfortunately visible to others.

I've been mentally preoccupied and worrying about my family since arriving in Grenada. It was difficult leaving my pregnant wife at home in Denmark with our 12-year-old son but we often Skype and I do as much of my usual household duties as possible online. I generally try to make sure there isn't much disruption to their daily routine because I'm not there.

My son and I enjoy playing chess together so we still do this but online. I read and give him tips for his essays using online tools like Google docs and upload photos of my work here as he's interested in this as well. My wife is scheduled to give birth shortly after I return and we are getting everything ready for when our new-born girl arrives. We often send each other links to products we want to buy for the baby and also other stuff we need for the home generally. I sometimes also surprise her with seasonal flowers and am still able to order these online. I continue to pay bills, do our banking online, and order pet food and other household essentials online as normal.

Survey questions

- **What potential difficulties do you foresee [Persona] having [PERCEIVING/UNDERSTANDING/OPERATING] Websites?** Consider what it must be like to experience a particular symptom and how that can impact your ability to do the things you want to do online. It may also help thinking about [Persona] using websites that you use. Be sure to make reference to the general and or specific aspects of websites that you foresee being difficult for [Persona]. You can also include links to examples of these aspects.

- **Why do you think [Persona] may encounter those difficulties?** Kindly attempt to address each difficulty and be as detailed as possible.
